# Biological and metabolic effects of the association between the microalga *Galdieria sulphuraria* and the fungus *Penicillium citrinum*

**DOI:** 10.1038/s41598-023-27827-6

**Published:** 2023-01-31

**Authors:** Maria Michela Salvatore, Federica Carraturo, Giovanna Salbitani, Luigi Rosati, Arianna De Risi, Anna Andolfi, Francesco Salvatore, Marco Guida, Simona Carfagna

**Affiliations:** 1 Department of Chemical Sciences, University of Naples Federico II, Naples, Italy; 2 Institute for Sustainable Plant Protection, National Research Council, Portici, NA Italy; 3 Department of Biology, University of Naples Federico II, Naples, Italy; 4 Hygiene Laboratory, Centro Servizi Metrologici e Tecnologici Avanzati (CeSMA), University of Naples Federico II, Corso Nicolangelo Protopisani, 80146 Napoli, NA Italy; 5 BAT Center - Interuniversity Center for Studies on Bioinspired Agro-Environmental Technology, University of Naples Federico II, Portici, NA Italy

**Keywords:** Chemical biology, Computational biology and bioinformatics, Plant sciences

## Abstract

Contamination of microalgae cultures can reduce their productivity and affect the quality of biomass and valuable bioproducts. In this article, after having isolated and identified for the first time the filamentous fungus *Penicillium citrinum* from heterotrophic cultures of the red polyextremophilic microalga *Galdieria sulphuraria*, we investigated the biological and metabolic significance of this alga-fungus association. In the same medium, both organisms grow better in each other's presence than separately. Both cell density and cell size of *G. sulphuraria* increase in co-cultures compared to pure alga cultures. In co-cultures, despite very severe growth conditions, the load of *P. citrinum* increases compared to pure fungus cultures. Optical microscope images have shown physical contact between cells of *P. citrinum* hyphae and *G. sulphuraria* which, however, retain their morphology and cell wall intact. GC–MS-based metabolomics analysis of metabolites excreted in the culture medium shows that pure cultures of the fungus and alga and co-cultures of alga plus fungus can be easily differentiated based on their metabolic products. Indeed, a richer assortment of extracellular metabolites (comprising both products of primary and secondary metabolism) is a distinct feature of co-cultures compared to both pure alga and pure fungus cultures.

## Introduction

Microalgae are essentially unicellular photoautotrophic organisms capable of colonizing any type of terrestrial habitat including extreme environments^1–3^. Phylogenetically, these organisms comprise many different groups, colonizing aquatic and/or terrestrial sites, and can grow over a wide range of temperatures, salinity, pH values, or different light intensities.

The red microalga *Galdieria sulphuraria* (Cyanidiaceae) is the dominant living form in sulfuric acid hot springs worldwide (pH 0.05–5.0, 35–56 °C)^4–6^. The genus *Galdieria* includes species capable of growing heterotrophically in the dark using different carbon sources as organic substrates such as sugars, polyols, amino acids and organic acids^5^. According to Barone et al.^7^ these microalgae can grow on about 50 different organic substrates.

In the last decade, microalgae have aroused considerable interest for the numerous biologically active compounds present in their biomass (e.g., proteins, polyunsaturated fatty acids, pigments, vitamins and minerals) or as extracellular compounds (e.g., oligosaccharides)^8,9^. Furthermore, the large-scale cultivation of microalgae has increased significantly in recent years due to their potential wide application in the renewable energy, bioremediation, food, biopharmaceutical and nutraceutical industries^9–12^.

One of the main problems in algae cultivation is the presence of biological contaminants^13^ which can affect cell growth and productivity, as well as the quality of the biomass^14^. Microorganisms that usually affect the growth of microalgae include bacteria, viruses, oomycetes, and fungi. Although some microalgae can thrive in very extreme habitats where other organisms hardly survive^6,12^, the proliferation of contaminants can also occur in severe environmental conditions.

Microalgae grown in heterotrophic conditions do not carry out photosynthesis but draw nourishment from other sources of organic carbon (such as glucose, glycerol, and sucrose) added to the culture medium. Obviously, the presence of organic substrates facilitates the proliferation of microbial communities (e.g., fungi, bacteria, viruses, and others) in the medium because these microbes generally grow faster than microalgae^15^. For some specific algae production purposes such as pharmaceuticals, cosmetics or food, the algal biomass cannot be contaminated.

Microalgae cultures can be affected by, inter alia, several species of fungi, some of which have not yet been fully identified^16,17^. Fungi usually grow at temperatures between 0 and 30 °C, but there are various species that can grow at temperatures close to 50 °C, or below 10 °C. Furthermore, fungi tolerate acidic media for growth even though pH ≈ 6 is more suitable for several species studied^18^. This information makes it clear that even the cultivation of extremophile microalgae (at temperatures above 30 °C or at very acidic pH) is not free from the risk of fungal contamination.

However, fungi often engage in mutualistic interactions with the host and may enhance the host's growth and fitness by aiding in nutrient acquisition and improving its ability to resist or adapt to abiotic and biotic stressors^19,20^. Mutualistic interactions between fungi and algae can also enhance the metabolism of both eukaryotic organisms and can lead to the production of interesting and valuable metabolites which, in many cases, have been isolated for biotechnological applications^21–23^. For example, Du et al.^24^ reported that the symbiosis between the fungus *Mortierella elongata* and the marine microalga *Nannochloropsis oceanica* led to the improvement of their specific metabolic pathways, with increased oil production, compared to pure fungus or algal cultures. Similarly, several studies have shown that, within what appears to be their contribution to host fitness, fungi can produce a variety of biologically active substances that help solve human, animal, and plant health problems (e.g., natural antimicrobials, insecticides, and anticancer agents) and which structures inspire the synthesis of valuable new compounds^25–30^. Several researchers have reported the isolation and characterization of fungi from different hosts^31–34^, but up to now there are no studies on the isolation of fungi from heterotrophic cultures of *G. sulphuraria*.

This study arises from the observation and isolation of a fungus from heterotrophic cultures of *G. sulphuraria* grown in the dark at 34 °C in a medium at pH 1.2 supplied with glucose. Molecular analysis identified the fungus as *Penicillium citrinum*.

Following this discovery, we undertook the study of pure cultures of *P. citrinum*, *G. sulphuraria* and co-cultures of both organisms to shed light on the biological significance and metabolic effects of their association.

First, the growth capacity of alga and fungus grown individually or together in the same culture medium was evaluated. Some optical microscope investigations have also made it possible to visualize the contacts between the two organisms. Finally, to investigate the metabolic interactions that may exist between *G. sulphuraria* and *P. citrinum*, we employed a GC–MS based metabolomics untargeted footprint strategy that leverages on the comparison of metabolites excreted in medium in pure cultures of *P*. *citrinum* or *G. sulphuraria* and co-cultures of both organisms.

## Results

### Algal growth

**Figure 1:**
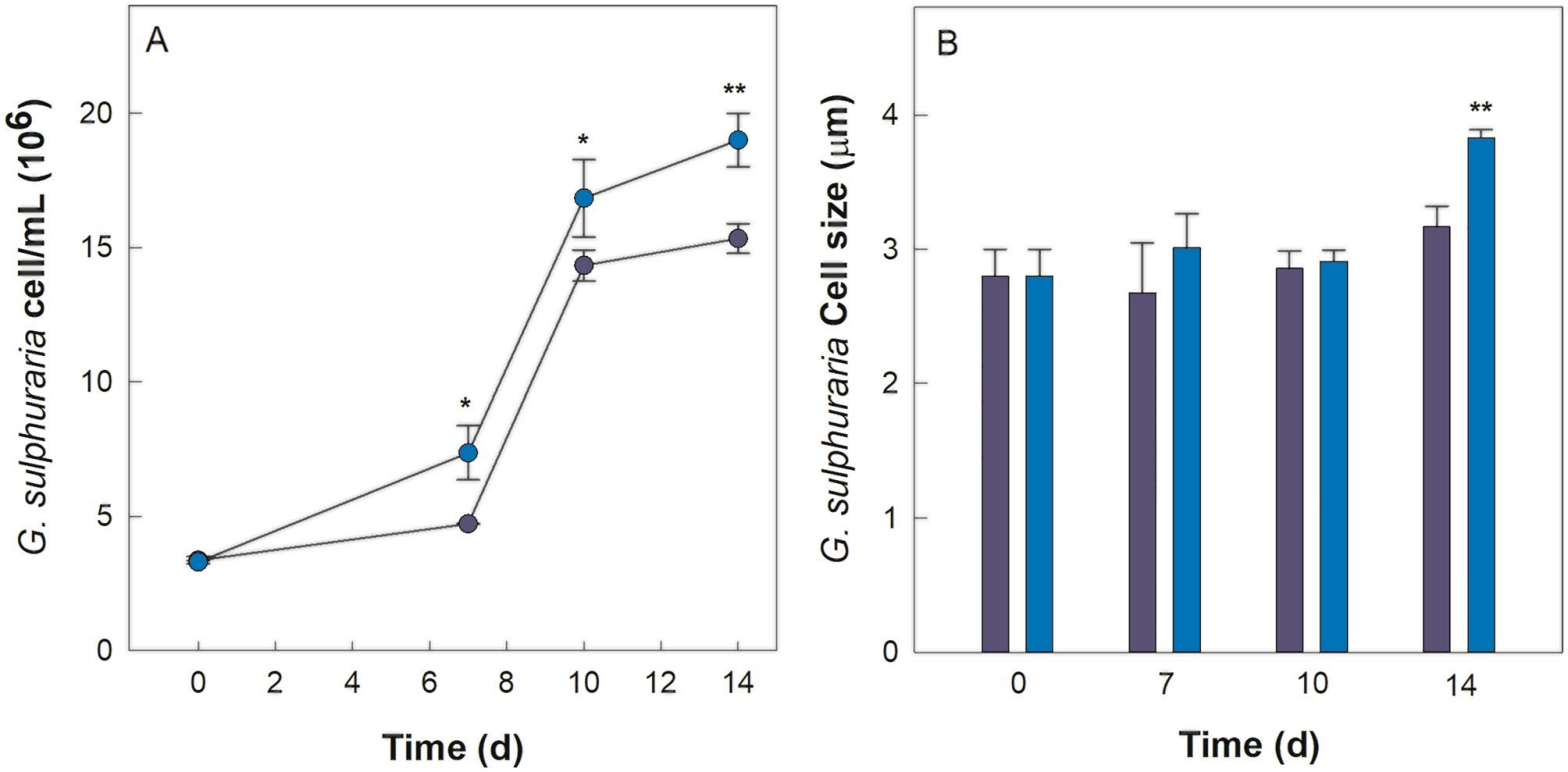
(**A**) Cell density of *Galdieria sulphuraria* grown in an axenic (violet) and in a co-culture with *Penicillium citrinum* (dark blue). (**B**) Average cell diameter of *G. sulphuraria* grown in an axenic and in a co-culture. Error bars represent SD (*n* = 3). Significant difference was determined by One-way ANOVA with post-hoc Tukey HSD Test (*p-value < 0.05, **p-value < 0.01). (d = days).

*Galdieria sulphuraria* growth was evaluated both in heterotrophic axenic cultures and in co-cultures with *P*. *citrinum* for a period of 14 days and results are exposed in Fig. 1.

As shown by Fig. 1A, the density (cells mL^−1^) of *G. sulphuraria* cells follows an increasing trend both in the pure alga cultures and in co-cultures of the alga with the fungus. This increasing trend continues up to 14 days when algal cell density is found to be significantly larger in co-cultures than in the pure algal cultures. Furthermore, Fig. 1B shows that average size (µm) of cells of *G. sulphuraria* from co-cultures is, at 14 days, significantly larger with respect to cells from pure *G. sulphuraria* cultures.

From this it can be deduced that *G. sulphuraria* growth is enhanced by the presence of *P*. *citrinum* in the same medium.

### Fungal growth

**Figure 2:**
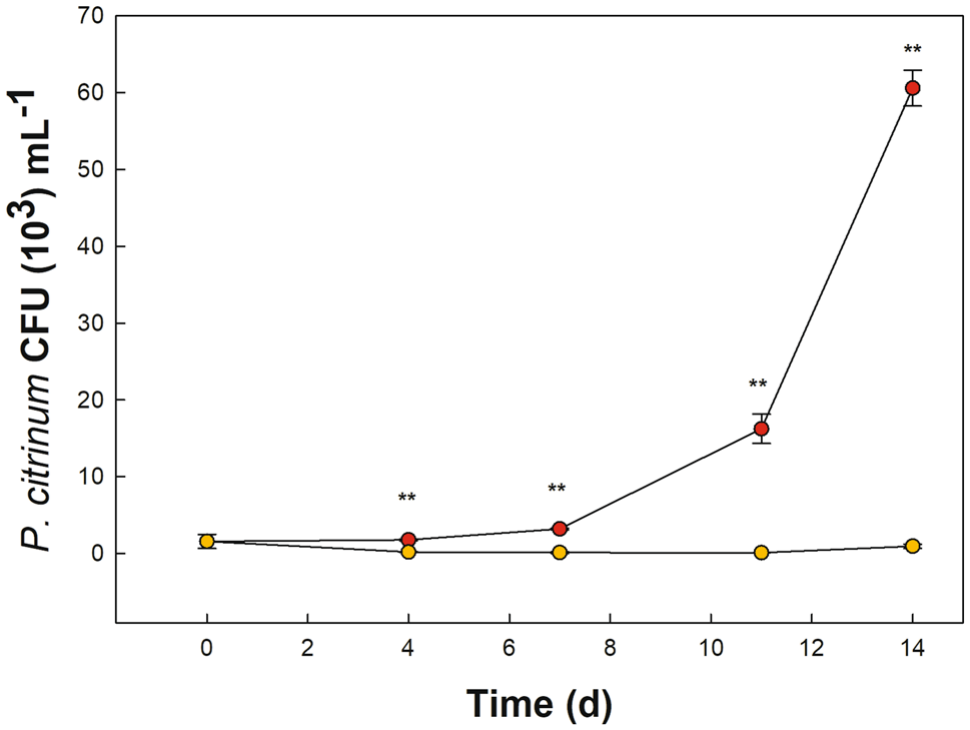
Microbial load of *Penicillium citrinum* cultured alone (yellow) and in a co-coltures with *Galdieria sulphuraria* (red). The microbial load was expressed as Colony Forming Unit per mL (CFU mL^−1^). At a significance treshold of 0.05, *P. citrinum* load is significantly larger in co-cultures starting at 10 d (d = days). Error bars represent standard deviation (*n* = 3). Significant difference was determined by One-way ANOVA with post-hoc Tukey HSD Test (**p-value < 0.01).

Growth curves of *P. citrinum* in the pure fungus cultures and co-cultures with *G. sulphuraria* are presented in Fig. 2. Very different trends for fungal growth curves in pure *P. citrinum* cultures and in co-cultures with alga can be observed.

The fungal growth curve for the pure fungus cultures seems to have a slightly decreasing trend which continues up to 14 days. After 14 days, nominally, this trend reduces the average fungus load from 2 × 10^3^ to about 10^2^ CFU mL^−1^.

On the contrary, the fungus load steadily increases in the co-cultures of *G. sulphuraria* and *P. citrinum*. After ten days, the fungus load in co-cultures is significantly greater than in the pure fungus cultures and, after 14 days, the fungus load in the co-cultures has reached about 6 × 10^4^ CFU mL^−1^ which is larger than the initial load for a factor of about 30.

From Fig. 2 there can be no doubt that *P. citrinum* takes advantage from the presence of *G. sulphuraria* in the same medium.

### Microscopy observation

**Figure 3:**
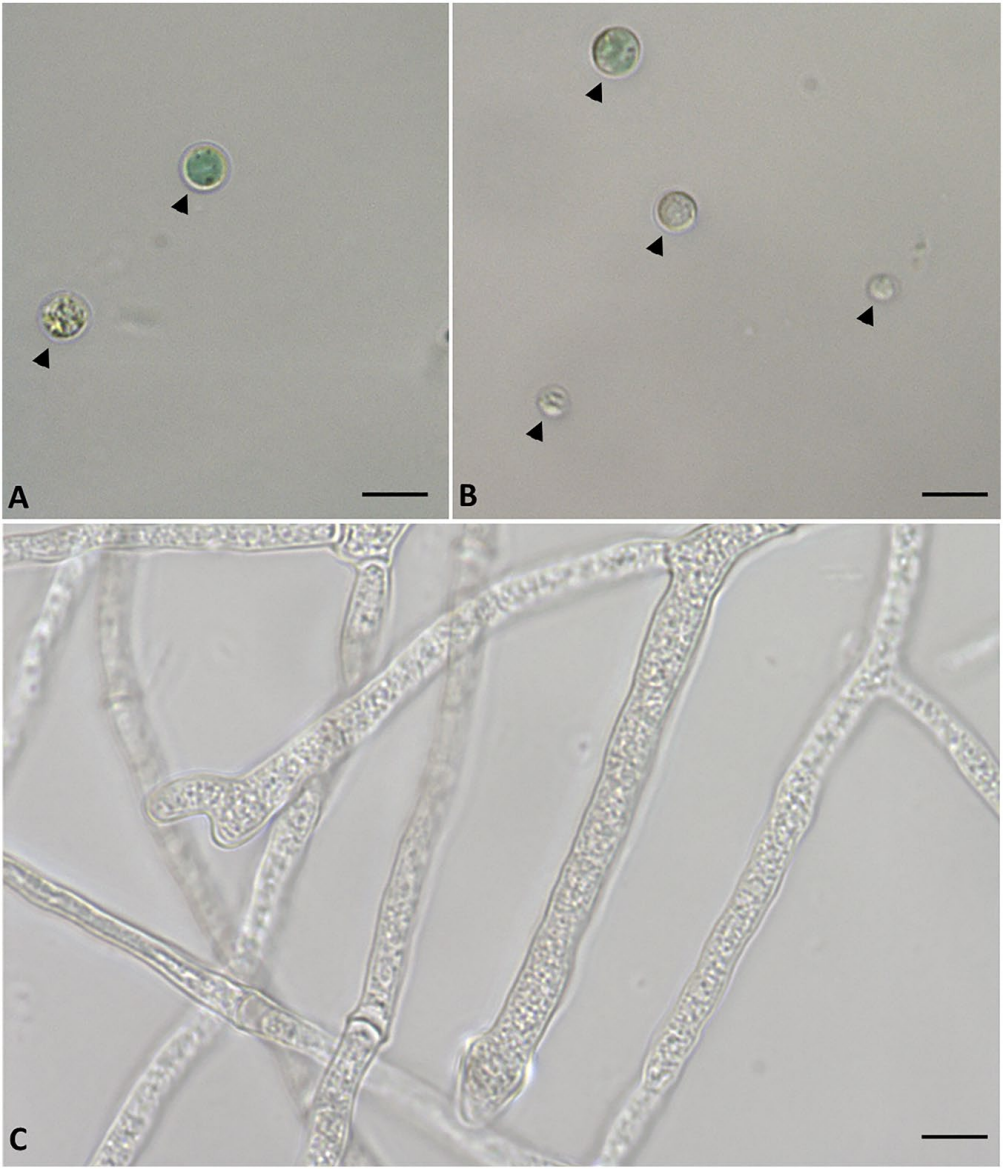
Images of the alga *Galdieria sulphuraria* in light microscopy. (**A**,**B**) Samples of alga in which are evident cells with a rounded shape, the cell wall is clearly visible (arrowhead). (**C**) samples of fungus *Penicillum citrinum* in which are evident fungal hyphae of different morphology. Bars correspond to 5 µm.

**Figure 4:**
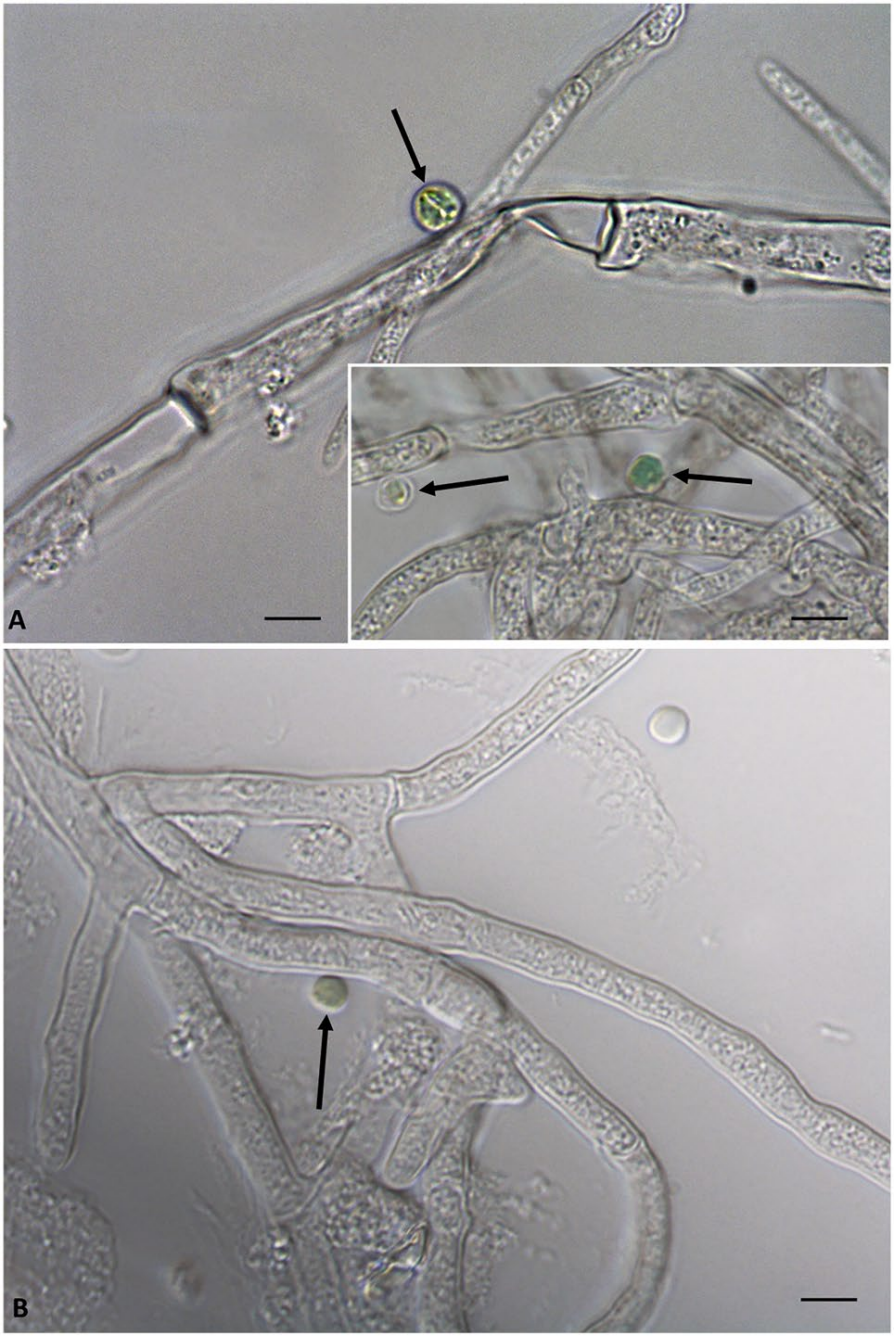
Images of relationships between the alga *Galdieria sulphuraria* and the fungus *Penicillum citrinum*. (**A**) Insert: Images of light microscopy of interaction between alga and fungus (arrow). (**B**) Images of phase contrast microscopy of associations between alga and fungus (arrow). Bars correspond to 5 µm.

Light microscopy observations were performed on samples of *G. sulphuraria* and *P. citrinum* pure cultures and in co-cultures, while phase-contrast microscopy observations were performed only on co-cultures samples. The results reported in Fig. 3A,B show in light microscopy the sample with algae where it is evident the round cells with regular morphology and cellular wall; while Fig. 3C shows the samples with the fungus in which the fungal hyphae with different morphology are evident. Differently, in Fig. 4 it is shown in light (Fig. 4A, insert) and in phase-contrast microscopy (Fig. 4B), the relationships existing between *G. sulphuraria* and *P. citrinum*. In details, the images show that on the fungal hyphae are deposited the alga cells with round and regular morphology. Furthermore, it is evident that the cellular walls of alga cells after interaction were not altered (Fig. 4A, insert, B).

### GC–MS analysis of extracellular metabolites

To investigate differences or similarities in the metabolism of the pure and co-cultures of *P. citrinum* and *G. sulphuraria*, extracellular metabolites excreted by microorganisms in the culture medium were investigated by a footprint untargeted metabolomic strategy. To this end, the cultural filtrates were extracted with EtOAc and submitted to GC–MS analysis, after derivatization with BSTFA. Overall, a dataset comprising 18 observations (GC–MS chromatograms) was collected.

The dataset incorporates three classes corresponding to the three culture conditions investigated. Each class is made up of six observations (GC–MS data files) representing technical and biological replicates under identical acquisition and culture conditions. The three classes correspond to co-cultures of *P. citrinum* and *G. sulphuraria*, identified by label (Alga + Fungus), and to pure cultures of *G. sulphuraria* or *P. citrinum* identified as pure (Alga) or pure (Fungus) cultures, respectively.

As reported in Methods, the GC–MS datafiles were processed with AMDIS^35^ and SpectConnect^36^ programs to create a Relative Abundance matrix (RA matrix), with columns corresponding to observations and rows to ‘conserved metabolites’, which will be the basis of the following evaluations.

#### Multivariate unsupervised data analysis

Agglomerative Hierarchical Clustering (AHC) of columns of the RA matrix is a suitable algorithm to compare observations in order to find naturally occurring groups in the data without using a priori information (unsupervised).

**Figure 5:**
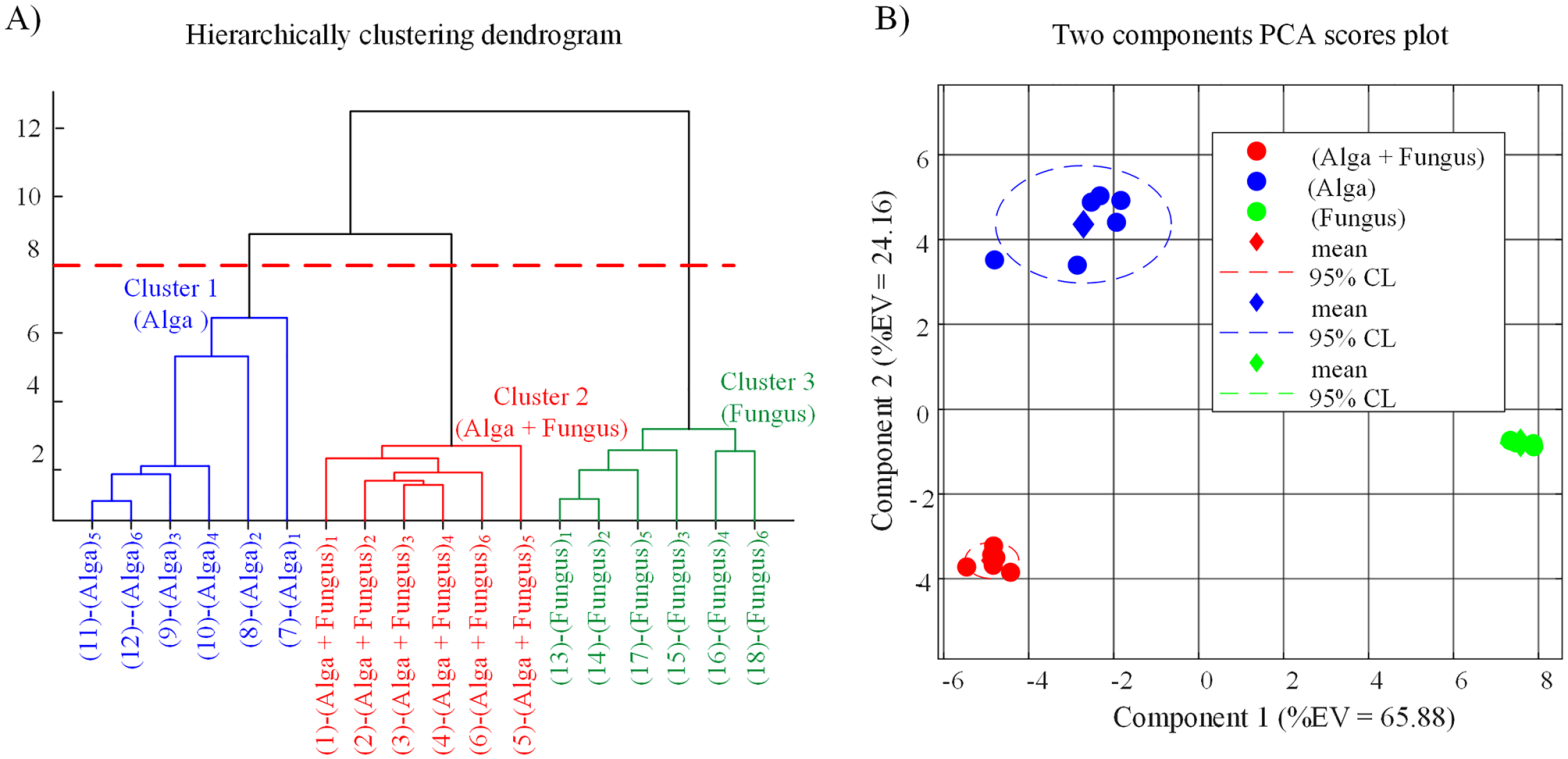
Graphical presentation of multivariate unsupervised data analysis: (**A**) Dendrogram obtained from hierarchically clustering columns of RA matrix with average linkage and Euclidean distance. The red broken line cuts the dendrogram at a height of 8. This cut results in three distinct clusters, shown in different colours. (**B**) Scores plot calculated by submitting observations in RA matrix to multiclass Principal Component Analysis (PCA). (Alga) = *Galdieria sulphuraria* pure cultures; (Fungus) = *Penicillium citrinum* pure cultures; (Alga + Fungus) = *Penicillium citrinum* and *Galdieria sulphuraria* co-cultures. EV = Explained variance.

The dendrogram which is obtained by applying the AHC algorithm to columns of the RA matrix is shown in Fig. 5A. From Fig. 5A, we easily deduce that the AHC algorithm groups observations into three clusters each cluster containing observations within one class. Furthermore, the three classes are linked to each other by highly inconsistent links which demonstrate that the three observations clusters exhibit a remarkable difference from each other.

These deductions are further confirmed by principal component analysis (PCA) of data in the RA matrix. In fact, although PCA is strictly a dimension reduction technique which allows visualization of multidimensional data, it is often used as an unsupervised clustering technique. PCA scores plots of the 18 observations in the RA matrix are shown in Fig. 5B. Even superficial inspection of Fig. 5B will show that it is perfectly consistent with the hierarchical tree in Fig. 5A.

In fact, observations pertaining to cultures of the fungus are strictly grouped and well separated both from observations in the classes (Alga + Fungus) and (Alga) along the first component axis. On the other side observations in the classes (Alga + Fungus) and (Alga) are more like to each other being well separated only along the second component axis. This is because the link connecting cluster (Alga + Fungus) to cluster (Alga), in the dendrogram of Fig. 5A, exhibits a lower degree of inconsistency compared to the link connecting cluster (Fungus) to the other two.

Treating the data with two unsupervised clustering algorithms, which operate on very different clustering principles, ensures that natural groupings in the data are found and artifacts are avoided.

#### Multivariate supervised data analysis

**Figure 6:**
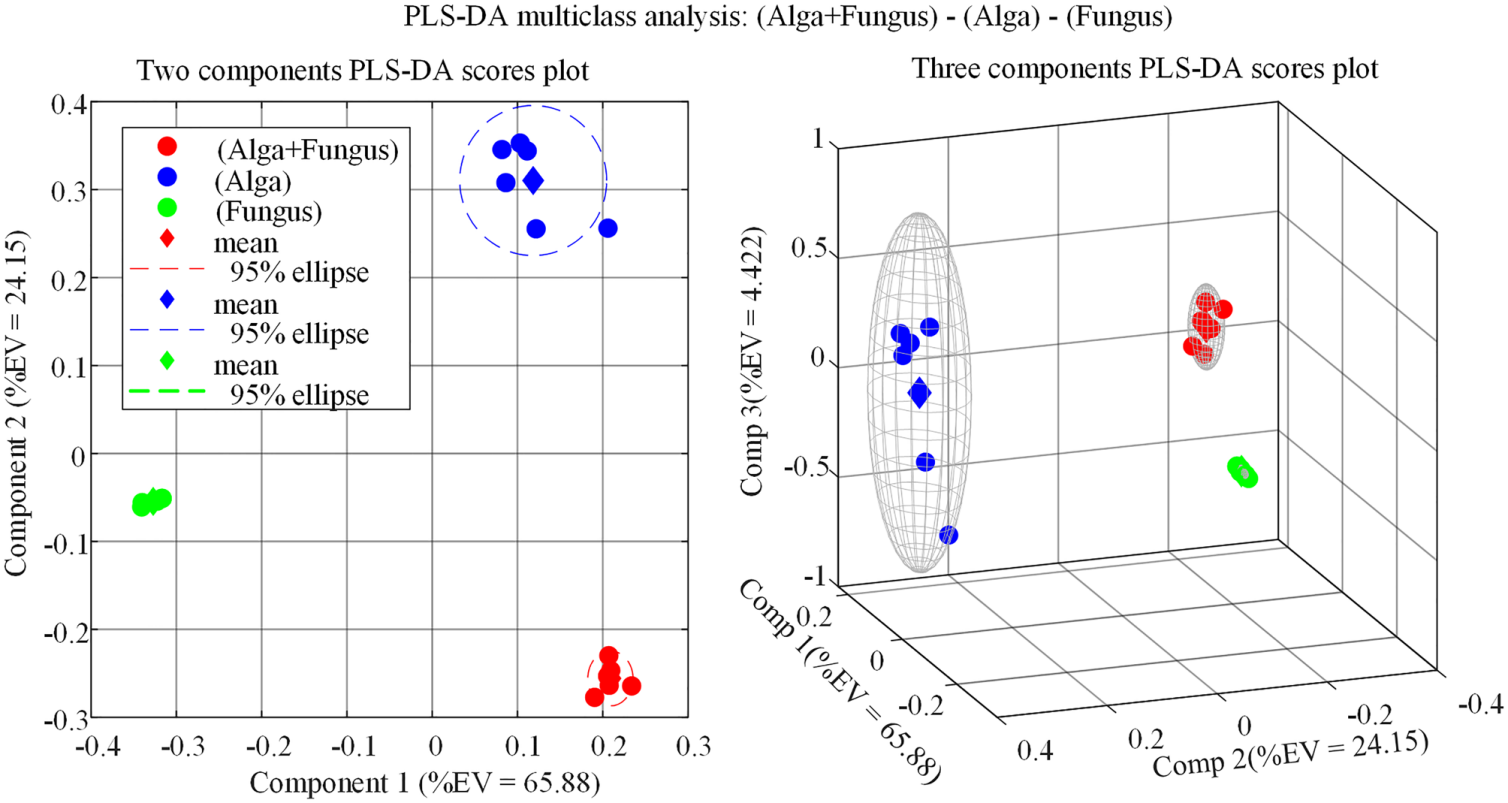
Partial Least Squares Discriminant Analysis (PLS-DA) of extracellular metabolites in cultures of (Alga + Fungus), (Alga) and (Fungus). (Alga) = *Galdieria sulphuraria* pure cultures; (Fungus) = *Penicillium citrinum* pure cultures; (Alga + Fungus) = *Penicillium citrinum* and *Galdieria sulphuraria* co-cultures. EV = Explained variance.

As a complement to unsupervised hierarchical clustering and PCA analysis in Fig. 5, partial least squares discriminant analysis (PLS-DA) is a very convenient supervised multivariate data analysis technique since it allows to rank variables (metabolites) based on their Variable Importance for Projection (VIP) score. In general, variables (metabolites) with VIP values close to or greater than 1 can be considered the most important for the PLS-DA model. The results of the PLS-DA analysis of the GC–MS dataset collected in this study are presented in Fig. 6 and Table 1.

**Table 1:** Results of multiclass PLS-DA analysis in Fig. 6.

Name	ID##	VIP scores	Coordinates
Unknown (RI = 1860)	(42)	1.276	{0 1 0}
CitraMalic acid, 3TMS (RI = 1491)	(18)	1.242	{1 0 0}
Uracil, 2TMS (RI = 1357)	(10)	1.241	{1 0 0}
Protocatechuic acid, 3TMS (RI = 1838)	(33)	1.235	{1 1 0}
Citric acid, 4TMS (RI = 1845)	(34)	1.228	{1 0 0}
Unknown (RI = 1705)	(30)	1.219	{1 0 0}
Unknown (RI = 1870)	(36)	1.213	{1 1 0}
Lactic Acid, 2TMS (RI = 1084)	(4)	1.175	{1 1 1}
α-Ketoglutaric acid, 3TMS (RI = 1623)	(26)	1.158	{1 1 0}
Unknown (RI = 1629)	(27)	1.150	{1 1 0}
Homogentisic acid, 3TMS (RI = 1855)	(35)	1.145	{1 0 0}
Unknown (RI = 1471)	(17)	1.133	{1 1 0}
Unknown (RI = 1392)	(13)	1.088	{1 0 0}
Tyrosol, 2TMS (RI = 1586)	(21)	1.085	{1 1 0}
Unknown (RI = 1368)	(39)	1.072	{1 0 0}
Unknown (RI = 1413)	(16)	1.069	{1 0 0}
Fumaric Acid, 2TMS (RI = 1355)	(11)	1.058	{1 1 0}
Unknown (RI = 1968)	(38)	1.031	{1 1 0}
*meso*-Erythritol, 4TMS derivative (RI = 1532)	(40)	1.018	{1 0 0}
2-Propenoic acid, 3-methoxy-3-[(trimethylsilyl)oxy]-, methyl ester (RI = 1192)	(7)	1.003	{1 1 0}
2-Hydroxyglutaric acid, 3TMS (RI = 1589)	(22)	1.002	{1 1 0}
Isobutyric acid, 2TMS (RI = 1172)	(6)	0.992	{1 1 0}
4-HydroxyphenylLactic acid, 3TMS (RI = 1922)	(37)	0.968	{1 0 0}
Unknown (RI = 1402)	(15)	0.961	{1 1 0}
Unknown (RI = 1384)	(12)	0.958	{1 1 0}
Arabinofuranose, 4TMS (RI = 1765)	(31)	0.951	{1 0 0}
4-Hydroxyphenylacetic acid, 2TMS (RI = 1653)	(29)	0.946	{1 1 0}
Unknown (RI = 1395)	(14)	0.930	{1 1 0}
Pyroglutamic acid, 2TMS (RI = 1542)	(20)	0.921	{1 1 0}
Glycerol, 3TMS (RI = 1287)	(8)	0.894	{1 1 1}
Talofuranose (RI = 1873)	(51)	0.889	{0 0 1}
d-Glucose, 5TMS (RI = 1925)	(52)	0.886	{0 0 1}
Isovanillin, TMS (RI = 1554)	(50)	0.885	{0 0 1}
Phosphoric acid, 3TMS (RI = 1289)	(41)	0.882	{0 1 0}
Glycolic acid, 2TMS (RI = 1091)	(44)	0.880	{0 0 1}
Stearic acid, TMS (RI = 2245)	(54)	0.880	{0 0 1}
Palmitic acid, TMS (RI = 2049)	(43)	0.875	{0 1 1}
Malic acid, 3TMS (RI = 1507)	(19)	0.874	{1 1 0}
Meglutol, 3TMS (RI = 1621)	(25)	0.867	{1 0 0}
Unknown (RI = 1643)	(28)	0.862	{1 0 0}
Acetin, 2TMS (RI = 1193)	(45)	0.861	{0 0 1}
2-IsopropylMalic acid, 3TMS (RI = 1597)	(23)	0.850	{1 1 0}
2,3-Butanediol, 2TMS (RI = 1065)	(3)	0.834	{1 1 0}
Succinic acid, 2TMS (RI = 1329)	(9)	0.814	{1 1 0}
Sorbic acid, TMS (RI = 1135)	(5)	0.773	{1 0 0}
Adipic acid, 2TMS (RI = 1515)	(49)	0.768	{0 0 1}
Unknown (RI = 1293)	(47)	0.763	{0 0 1}
Boric acid, 3TMS (RI = 1021)	(1)	0.636	{1 1 1}

Identified metabolites are sorted according to descending VIP scores. The array in column labelled ‘Coordinates’ indicates ordinately the class membership of metabolites:(Alga + Fungus) = {1 − −}; (Alga) = {− 1 −}; (Fungus) = { − − 1}. RI represents Kovats retention index and TMS is the trimethylsilyl function, (CH_3_)_3_Si-.

The PLS-DA model in Fig. 6 was built by using three PLS-DA components and validated by the statistics: R2X = 0.944; R2Y = 0.994; Q2Y = 0.983. The high value of Q2Y, calculated using mean squared errors from fivefold cross-validation, indicates that the PLS-DA model is a predictive one.

It is easy to see, by comparing Figs. 5B and 6, that model from PLS-DA analysis is very similar to model from PCA analysis, even though PLS-DA is a classification technique in which class information is known ex ante and used to develop the model.

Apart from that, from Table 1 we see that 34, out of 48 listed ‘conserved metabolites’, were identified by their mass spectrum and temperature programmed Kovats retention index (RI). The 14 ‘conserved metabolites’ that could not be identified are listed as ‘Unknown’ in Table 1. In an untargeted approach, the GC–MS signals of unidentified metabolites are treated exactly like the signals of identified metabolites because they may participate in the discrimination between different conditions. However, since they are known only through their RI and mass spectrum (MS) and cannot be linked to specific substances, any consideration of their biological role is hampered.

In Table 1, in addition to the VIP score deduced from the PLS-DA analysis in Fig. 6, an ID (##) is associated with each metabolite, which will be used later as a substitute for its full name.

The last column of Table 1 contains, for each metabolite, a 1 × 3 array of logical digits (0 = absent, 1 = present) which we will call the ‘metabolite coordinates in the space of classes’. The three coordinates of a metabolite define its class membership. The first coordinate defines membership to class (Alga + Fungus), the second coordinate membership to class (Alga) and the last coordinate represents membership to class (Fungus). For example, the coordinates of the metabolite with ID (34) (corresponding to citric acid) are {1 0 0}; this implies that citric acid was detected only in extracts from cultures (Alga + Fungus); on the opposite side, the metabolite with ID (4) (corresponding to lactic acid) was detected in all samples since its coordinates are {1 1 1}.

By adding the first, second and third coordinates over the complete set of 48 metabolites, we obtain, respectively, the total number of metabolites detected in cultures (Alga + Fungus), (Alga) and (Fungus). The co-cultures (Alga + Fungus) are the richest and the cultures (Fungus) are the poorest in metabolites. Indeed, as visually shown in Fig. S1, Table 1 exposes 37 metabolites in class (Alga + Fungus), 26 metabolites in class (Alga) and 12 metabolites in class (Fungus).

Within a technical metabolomic frame, the coordinates associated with a given metabolite are of great value because they can be used to infer whether this metabolite is produced by the alga, the fungus, or both. For example, tyrosol (ID (21)) has coordinates {1 1 0} which means that it was detected only in classes (Alga + Fungus) and (Alga) and from this we can deduce that tyrosol is produced by the alga. Furthermore, if a metabolite has coordinate 1 (present) in a first class but coordinate 0 (absent) in a second class, it automatically means that this metabolite is upregulated in the first class compared to the second. For example, α-ketoglutaric acid (coordinates {1 1 0}), is upregulated in (Alga + Fungus) and (Alga) classes compared to (Fungus) class. Metabolites that are present in a single class represent a signature for the class in which they are detected. For instance, citric acid (coordinates {1 0 0}) and adipic acid (coordinates {0 0 1}) are signatures respectively for classes (Alga + Fungus) and (Fungus).

VIP score attributed to each metabolite in Table 1 can be interpreted as a measure of the contribution a metabolite makes to the ability of PLS-DA model to discriminate between classes. Because discrimination between classes depends fundamentally on metabolites which have significantly different levels in different classes, metabolites with high VIP scores tend to be unequally distributed between classes while metabolites uniformly distributed between classes tend to have low VIP scores^37^. For instance, consider protocatechuic acid which is upregulated in classes (Alga + Fungus) and (Alga) compared to (Fungus) cultures due to its coordinates {1 1 0}; protocatechuic acid must have significantly different levels in cultures of (Alga + Fungus) and (Alga) due to its high VIP score (1.235) indicating that protocatechuic acid strongly contributes to the discrimination between the three classes and it would not be so if it had not significantly different levels in cultures in which it is detected. In fact, from our data it results that protocatechuic acid is upregulated in class (Alga) compared to class (Alga + Fungus), as it is shown in the next paragraph. Next, consider two metabolites with coordinates {1 1 1} in Table 1 which, although present in all classes, have substantially different VIP values (e.g., lactic acid (VIP = 1.17), boric acid (VIP = 0.64)). On the base of the above criterion, we may infer that lactic acid (which has VIP value larger than 1) has significantly different levels in the three classes while boric acid (which has a VIP value substantially lower than 1) is homogeneously distributed between classes. This prediction is correct in this specific case as we can easily verify from the source data in the RA matrix. However, in making these predictions it must be kept in mind that the VIP parameter is a constrained indicator since the sum of the squares of the VIPs of all variables is equal to the number of variables (as can be verified from VIP values in Table 1 for which we calculate ∑VIP^2^ = 48). Indeed, this is the reason because it is common to consider most significant for the model variables with VIP value larger than 1 (i.e., larger than the average of squared VIP values). Thus, although a larger than average VIP value is unambiguously associated with an uneven distribution of the metabolite (e.g., lactic acid), a low VIP value may sometimes be due to the fact that the available amount of VIP within the model has been consumed by other metabolites. Indeed, the nature of the VIP calculation is such that if the model is rebuilt by progressively excluding variables with low VIP scores, new variables will always be below threshold and even metabolites not uniformly distributed between classes may progressively acquire low VIP values.

#### Univariate data analysis and pairwise comparisons

**Figure 7:**
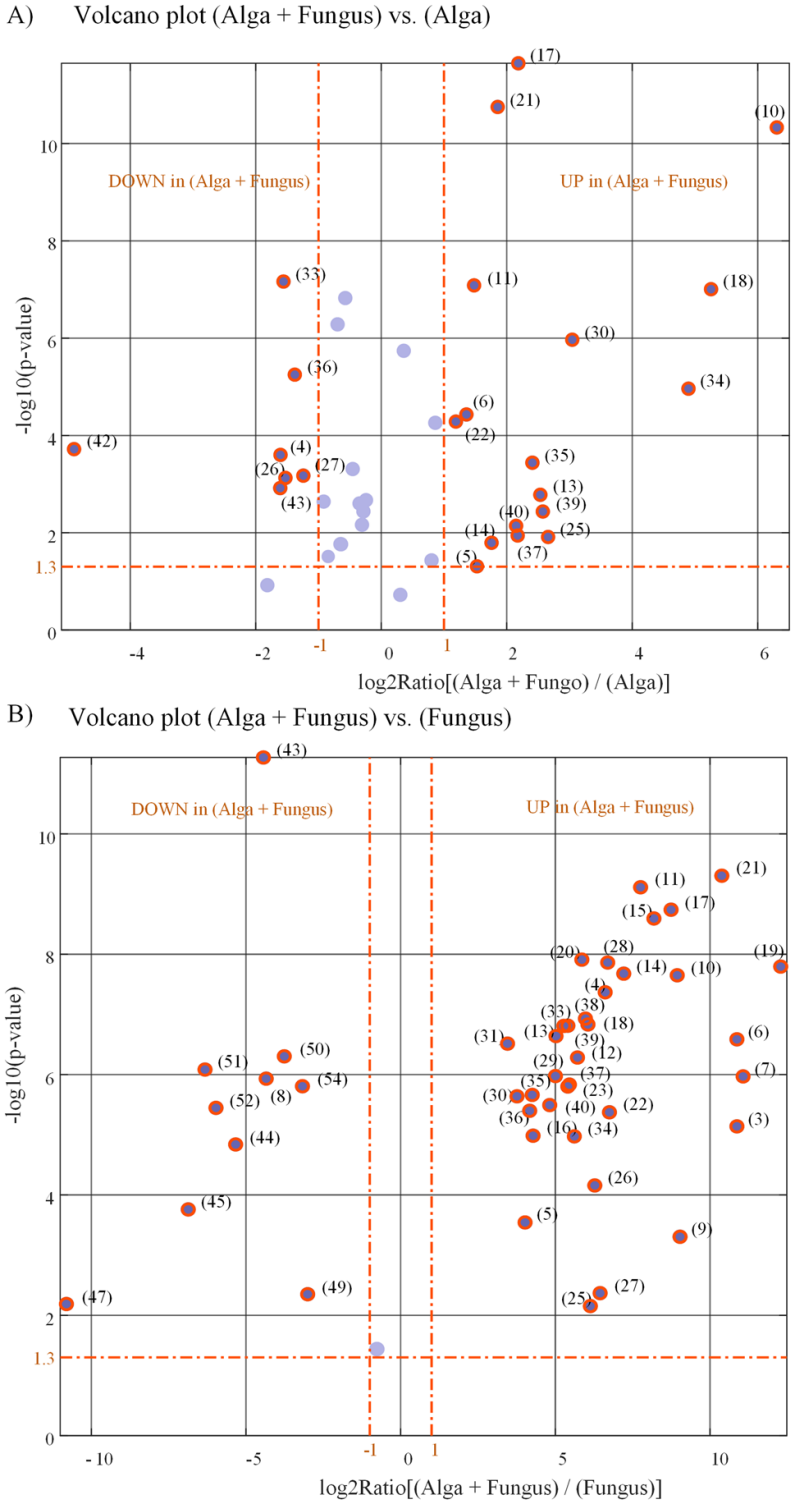
Results of univariate comparisons of: (**A**) (Alga + Fungus) vs. (Alga) classes and (**B**) (Alga + Fungus) vs. (Fungus) classes. For each metabolite, fold change (FC) is defined as the ratio between mean relative abundances in the compared classes. Only metabolites with *p*-value < 0.05 and fold changes greater than 2 or lower than 0.05 are considered to be differentially expressed in the two compared classes (orange rings). Blue dots represent metabolites with no significantly different levels in the two compared classes. Metabolites can be tracked back to identified metabolites in Table 1 through the ID(##) associated with each point in the volcano plot.

Univariate analysis of data was performed to compare, one by one, metabolites levels in any pair of classes (conditions). Results of the univariate comparison of the (Alga + Fungus) and (Alga) classes, for the 40 identified metabolites which are conserved in at least one of the two classes, are exposed in Fig. 7A (and in more detail in Table S2 and Fig. S2). Please note that metabolites with coordinates {0 0 1} which have been detected only in the pure fungus cultures are not considered in Fig. 7A.

As can be seen from the volcano plot in Fig. 7A, univariate analysis associates to each metabolite a fold change (FC) which is defined as the ratio between the average of the 1 × 6 vector of relative abundances representing the metabolite in the (Alga + Fungus) class and the average of the 1 × 6 vector of relative abundances representing the metabolite in the (Alga) class.

However, the calculated fold changes need to be validated for statistical significance. This has been done by associating, to each calculated fold change, a *p*-value which is calculated by submitting the two 1 × 6 vectors of relative abundances, from which the FC is calculated, to a Student’s t-test for equal means. Only metabolites with *p*-value < 0.05 and fold changes greater than 2 are considered to be upregulated in the (Alga + Fungus) class, and only metabolites with *p*-value < 0.05 and fold changes lower than 0.5 are considered to be upregulated in the (Alga) class (or downregulated in the (Alga + Fungus) class).

While the fold change calculation is straightforward for metabolites, which are conserved (detected) in both compared classes, problems are encountered for metabolites, which are observed only in one of the two classes. This is because one of the two compared vectors is a vector of all zeros so that an infinite (Inf) or zero fold change, and a point with a ± Inf abscissa in the volcano plot, would be obtained. This difficulty can be prevented by a data imputation strategy implemented on the RA matrix. A reasonable data imputation strategy might consist in substituting all zeros in a column of the RA matrix with a fraction of the minimum relative abundance value in the column. This is equivalent to attribute, to the undetected peak of an expected metabolite in a chromatogram, an area equal to a fraction of the area of the smallest peak detected.

Apart from that, from the volcano plot of Fig. 7A among the 40 metabolites which are conserved in at least one of the two classes, 16 have comparable levels in both compared classes. A large fraction of metabolites, 17, appears to be upregulated and only 7 metabolites are downregulated in the co-cultures (Alga + Fungus).

The most abundant metabolites in extracts from (Alga + Fungus) and (Alga) cultures are succinic acid and malic acid. In GC–MS chromatograms from extracts of the (Alga + Fungus) and (Alga) cultures, succinic acid accounts, on average, for 16.4% and 12.4%, respectively, of the Total Ion Current (TIC). Analogously, malic acid, on average, accounts for 7.9% and 5.7% of the TIC in chromatograms from co- and pure alga cultures extracts, respectively. Both succinic acid and malic acid are almost absent in extracts from pure fungus cultures.

However, in extracts from co- and pure alga cultures also the level of lactic acid is found to be relatively high. In fact, the lactic acid peak accounts, on average, for 9.3% and 26% of TIC in chromatograms from extracts of (Alga + Fungus) and (Alga) cultures, respectively.

**Figure 8:**
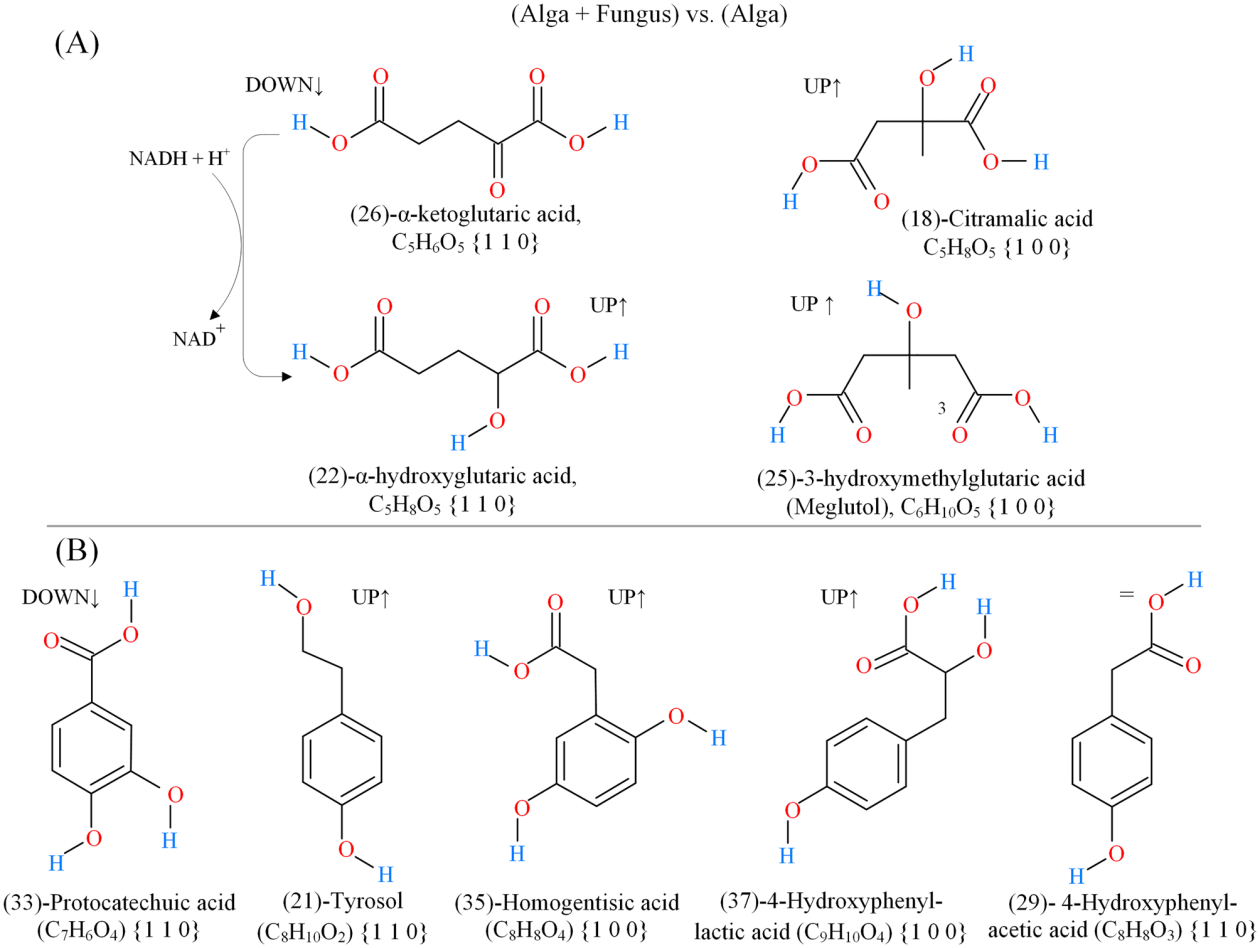
Structurally related metabolites detected in extracts from (Alga + Fungus) and/or pure (Alga) cultures which contribute to differentiate extracts from the diverse cultural conditions. (**A**) Compounds structurally related to α-hydroxyglutaric acid; (**B**) Phenolic compounds.

The content of Fig. 7A is further expanded in Fig. 8 which presents two groups of structurally related metabolites which contribute to the differentiation between (Alga + Fungus) and Alga classes. In particular, phenolic compounds in Fig. 8B appear to be secondary metabolites produced by the alga because they are not detected in the (Fungus) class as indicated by their {1 1 0} or {1 0 0} coordinates.

As it can be seen from the volcano plot in Fig. 7B (and in more detail from Table S3 and Fig. S3), the scenario is quite different when metabolites in extracts from (Alga + Fungus) cultures are compared to metabolites in extracts from (Fungus) cultures.

All the 46 metabolites (except boric acid), which are conserved in at least one of the two compared classes, are significantly different in the two conditions. In particular, 35 metabolites are upregulated in (Alga + Fungus) class and 10 metabolites are upregulated in (Fungus) class. The large number of upregulated metabolites in the co-cultures with respect to the pure fungus cultures is an obvious consequence of the fact, shown in Fig. S1, that a richer assortment of compounds is detected in the co-cultures. Furthermore, this is in accordance with results of above multiclass multivariate analysis which shows that observations in the (Alga + Fungus) class are much farther apart from observations in (Fungus) class than from observations in the (Alga) class (see Figs. 5 and 6).

## Discussion

In this study, we have investigated on the biological and metabolic significance of the association of the filamentous fungi *P. citrinum* with the polyextremophilic microalga *G. sulphuraria*.

Both *G. sulphuraria* and *P. citrinum*, when combined, exhibited improved growth. After 14 days of co-culture (Alga + Fungus), cell size as well as cell density of *G. sulphuraria* were significantly larger than that of the alga grown alone and *P. citrinum* load was greater than the load in the pure fungus cultures. Observations under the microscope showed a physical contact between fungus hyphae and the microalgal cells. Furthermore, the number, quantity and variety of detected metabolites in co-cultures of alga and fungus are higher than those produced individually by *G. sulphuraria* and *P. citrinum*.

This let us suppose a beneficial effect for both, due to the presence of the other.

As noted above, the most abundant metabolites in extracts from co-culture and pure alga cultures were succinic acid, malic acid and lactic acid. The implication of this fact is that the tricarboxylic acid cycle (TCA) and mitochondrial respiration are very active both in the co- and pure alga cultures as expected under aerobic conditions. Thus, to produce pyruvate to feed the TCA cycle, a very high flux through glycolysis must be maintained which, because of the non-cyclic nature of the glycolytic pathway, may result in depletion of cytosolic NAD^+^. Conversion of pyruvate to lactic acid, and its excretion to the medium, is the fastest way in which regeneration of cytosolic NAD^+^ can occur allowing glycolysis to continue^38,39^. This is a plausible explanation for the observed high level of lactic acid (which is not expected under aerobic conditions) in the pure alga and in co-cultures.

As can be seen from Fig. 8A, α-ketoglutaric acid and α-hydroxyglutaric acid form a redox couple and several NADH dependent enzymes (e.g., isocitrate dehydrogenase and lactate dehydrogenase) are known to be capable of the interconversion α-ketoglutarate ⇋ α-hydroxyglutarate^40–42^.

Reduction of α-ketoglutarate appears most effective in the co-cultures because α-ketoglutarate is downregulated and α-hydroxyglutaric acid is upregulated with respect to the pure alga cultures. It has been suggested that the function of α-hydroxyglutaric acid may be that of providing cells with a reservoir of reducing equivalents and that α-ketoglutarate ⇋ α-hydroxyglutarate interconversion may buffer cells redox potential^42^.

Two others hydroxy-dicarboxylic acids (i.e., meglutol and citramalic acid), which are structurally related to α-hydroxyglutaric acid, are also reported in Fig. 8A. As can be seen from their {1 0 0} coordinates, meglutol and citramalic acid are only detected in the co-cultures.

It is known that in methanogenic archaea citramalic acid is produced by direct condensation of pyruvate with acetyl-CoA, a reaction catalysed by a citramalate synthase protein (CimA)^42^. Bioproduction of citramalate has recently received much attention because it can be easily converted (by decarboxylation along with dehydration via an inexpensive hot pressurized water process) to methacrylic acid, a valuable precursor for the sustainable production of polymethacrylates^43^.

Finally, meglutol is a well-established human and plant metabolite known for its capability to inhibit hydroxymethylglutaryl-CoA reductase, the rate limiting enzyme in cholesterogenesis, which catalyzes the reduction of hydroxymethylglutaryl-CoA to mevalonic acid^44^. As far as it is known, meglutol is probably released by hydrolysis of 3-hydroxy-3-methylglutaryl-CoA, an intermediate in the degradation path of branched-chain amino acids, the ordinary fate of which is the conversion to acetyl-CoA and acetoacetate by hydroxymethylglutaryl-CoA lyase^45^.

Figure 8B exposes several phenols, which contribute to the differentiation of (Alga + Fungus) from (Alga) class. These metabolites have coordinates either {1 1 0} or {1 0 0} which means that they are not detected in the culture of the pure fungus, and, from that, we may infer that they are secondary metabolites produced by the alga. In the pure culture of the alga only protocatechuic acid, tyrosol and 4-hydroxyphenylacetic acid are detected while all of them are detected in the co-culture. In particular, in the co-culture, protocatechuic acid is downregulated with respect to the pure alga culture while tyrosol, homogentisic acid and 4-hydroxophenyllactic acid are upregulated. No change in the level of 4-hydroxyphenylacetic acid is observed. Although it is possible to speculate that the fungus consumes protocatechuic acid produced from the alga, we suggest that a decrease of the level of protocatechuic acid and an increase in the level of tyrosol, homogentisic acid and 4-hydroxophenyllactic acid represents an effect on the secondary metabolism of the alga induced by the presence of the fungus. Phenolic compounds are important products of the secondary metabolism and their production in different species of microalgae varied according to diverse characteristics of the culture medium such as temperature ranges, pH, nutrient composition and eventually the presence of extra carbon^46^. In *G. sulphuraria* the growth medium pH seems to have significant influence on the accumulation of phenolic compounds^47^. Since many phenolic compounds derived from different microalgal species demonstrated strong antioxidant and antimicrobial activity^48,49^, identification of these compounds in co-culture of *G. sulphuraria* and *P. citrinum* could have interesting implications for application purposes.

Finally, citric acid, which is detected only in the co-cultures, is another important factor that helps to differentiate the co-cultures from the pure alga and pure fungus cultures. Citric acid is a TCA intermediate which provides a bridge between TCA cycle and fatty acids metabolism. In fact, citrate can be exported from the TCA cycle to the cytosol and once in the cytosol it is broken down to acetyl-CoA and oxaloacetate. In the cytosol acetyl-CoA is further processed to malonyl-CoA which can be incorporated into fatty acids^50^. In plant cells and in microalgae, the citric acid exported from the mitochondria can be also isomerized to isocitrate and then decarboxylated to α-ketoglutarate, required by the plastids for nitrogen assimilation^51^. From our data it results that citric acid manifests itself in the co-cultures extracts while, contextually, palmitic acid (which has coordinates {0 1 1} and which is detected both in the pure alga and the pure fungus cultures) and stearic acid (which has coordinates {0 0 1} and which is detected in the pure fungus cultures) fade out. Thus, we suggest that while in the pure alga and pure fungus cultures citrate is withdrawn from the TCA cycle and converted into fatty acids, which are excreted to the medium, in the co-cultures production of fatty acids is fully inhibited or mitigated and excess citrate is excreted to the medium.

In conclusion, this study reveals for the first time the growth of the fungus *P. citrinum* in heterotrophic cultures of the polyextremophilic alga *G. sulphuraria*, despite very severe growing conditions (pH 1.2) for a fungus. Both organisms grow better in the presence of each other as demonstrated by number of cells of *P*. *citrinum* and by the number of cells and cell size of *G. sulphuraria* in co-cultures compared to pure alga and pure fungus cultures. This is in full agreement with metabolomic analysis of extracellular metabolites which can easily differentiate co-cultures, which are richer in both primary and secondary metabolism products, from pure cultures of the microorganisms. Several phenols (i.e., protocatechuic acid, tyrosol, homogentisic acid, 4-hydroxophenyllactic acid and 4-hydroxyphenylacetic acid) have been identified in co-cultures which may be interesting bio producible ingredients for the pharmaceutical or cosmetic industries.

## Methods

### Algal strains and inocula preparation

*Galdieria sulphuraria* cells strain 074G, (from the ACUF collection of the Department of Biology of the University of Naples “Federico II”) was employed for preparation of axenic algal inocula. Algal cultures were prepared in Allen medium^52^, containing 1.32 g L^−1^ (NH_4_)_2_SO_4_ and 2% (*w*/*v*) glucose, and grown at light (150 µmol photons m^2^ s^−1^) at 34 ± 1 °C in a thermostatic chamber (ANGELANTONI CH 770). All the flasks were stirred by an orbital shaker (120 strokes min^−1^) and were sparged with air (~ 80–100 L h^−1^) to create aerobic conditions.

### Fungus isolation, molecular characterization and inocula preparation

*Penicillium citrinum* was isolated from an accidentally contaminated heterotrophic culture of *G. sulphuraria* strain 074G.

The isolation of fungi from contaminated *G. sulphuraria* culture was performed according to ISO 21527-1:2008 protocol^53^, following a pre-enrichment step, consisting of incubating 10 mL of *G. sulphuraria* culture in 20 mL Sabouraud Dextrose Broth (Oxoid, Thermo Fisher Scientific Inc., Waltham, MA, USA) for 48–72 h at 22–25 °C. Aliquots of 1 mL were pour plated on Rose-Bengal chloramphenicol agar (DRBC, Oxoid, Thermo Fisher Scientific Inc., Waltham, MA, USA); Rose-Bengal agar plates were incubated for 72 h at 22 ± 1 °C. Different morphology colonies were sub-cultured in fresh Rose-Bengal agar and underwent molecular analysis^54^.

DNA extraction was performed to amplify and subsequently sequence fungal genomes, targeting fungal 18S rRNA gene. For samples under analysis, directly starting from fungal colony, 2 g mycelium from Rose-Bengal selective agar plates were aliquoted and centrifuged to recover DNA from the supernatant^54,55^. Supernatant was transferred into sterile 2 mL tubes containing 500 mg glass beads. CTAB extraction protocol^56–58^ was carried out to recover fungal DNA. The DNA samples were amplified with PCR, using a TECHNE Prime Thermal Cycler, for the characterization of fungi, employing ITS1 forward (5′-GGA AGT AAA AGT CGT AAC AAG G-3′ 5’-TCC GTA GGT GAA CCT GCG G-3’) and ITS4 reverse (5′-TCC TCC GCT TAT TGA TAT GC-3′) primers (Biofab Research, Rome, Italy) complementary to ITS-5.8S rDNA region of the fungal 18S rRNA gene (700 bp)^59^.

Sequencing reactions were performed by an external service (Biofab Research, Rome, Italy); results were then interpreted using an editing tool, Chromas Lite v. 2.6.6 (Technelysium Pty Ltd, South Brisbane, Australia). The identification of the isolates was conducted using BLASTN ver. 2.2.29 (also referring to GenBank), selecting the identity holding the highest percentage of identity with a 95% cut-off and a minimum E-value lower than E − 4^60^.

According to the Sanger Sequencing analysis and comparison with sequences available on BLASTIN database, the microorganism was classified as *Penicillium citrinum* strain SCAU200 with a 100% identity value (Max Score = Total Score = 944; Query cover = 96%; e-value = 0.0; Identity = 100.00%; Accession n. = MK281570.1).

For inocula preparation, the isolated fungal strain (*P. citrinum* strain SCAU200) was cultured in Sabouraud liquid medium, supplemented with chloramphenicol (Oxoid, Thermo Fisher Scientific Inc., Waltham, MA, USA). After 3 days cultivation, aliquots of 100 µL fungal culture were pour plated in Rose-Bengal chloramphenicol agar (DRBC, Oxoid, Thermo Fisher Scientific Inc., Waltham, MA, USA); agar plates were incubated for 72 h at 22 ± 1°C^53^. Starting from the cultured plate, an inoculum of *P. citrinum* at a concentration of 10^8^ CFU mL^−1^ was prepared in 40 mL of 0.1% NaCl in sterile 50 mL tubes kept under constant stirring at 200 r min^−1^. Cells concentration (CFU mL^−1^) of the microorganism was obtained by measuring absorbance at 600 nm with a Hach Lange DR6000 spectrophotometer (Hach Lange, Düsseldorf, Germany). At 600 nm an OD of 0.125 corresponds to a reference concentration of 10^7^ CFU mL^−1^. Inocula with a fungus concentration of 2 × 10^6^ CFU mL^−1^ (to be used for preparation of final cultures of *P. citrinum* and its co-cultures with the alga) were prepared from the 10^8^ CFU mL^−1^ inoculum by dilution with 0.1% NaCl.

### Preparation of cultures, growth conditions and growth analysis

Three different culture types (i.e., *G. sulphuraria* 074G pure culture; P. *citrinum* pure culture; co-culture of the alga and fungus) have been prepared in 1 L Allen Medium (pH 1.2), containing 1.32 g L^−1^ (NH_4_)_2_SO_4_, as nitrogen source, and 2% (*w/v*) glucose, as carbon source, for heterotrophic growth. Each culture type was prepared in triplicate in sterile Erlenmeyer flasks (2000 mL) and incubated in the dark for 14 days at 34 ± 1 °C. All the flasks were stirred by an orbital shaker (120 strokes min^−1^) and were sparged with air (~ 80–100 L h^−1^) to create aerobic conditions.

Experiments were performed with heterotrophic cultures of *G. sulphuraria*. Heterotrophic cells were prepared from autotrophically grown microalgal cells^61^ with an initial OD_800_ of ~ 0.2. After a period of adaptation (15 days), the cells were considered under heterotrophic condition.

Cultures of fungus and co-cultures were inoculated with 1 mL of inocula prepared to contain *P. citrinum* at a concentration of 2 × 10^6^ CFU mL^−1^. Thus, the final concentration of *P. citrinum* in the culture was 2 × 10^3^ CFU mL^−1^ in the final volume of 1 L (1 mL 2 × 10^6^ CFU mL^−1^ in 1L).

The cultures were sampled periodically, and algal growth was followed by measuring the number of cells by a Countess II FL Automated Cell Counter (Thermo Fisher Scientific).

To verify the growth of the fungus with or without *G. sulphuraria*, microbiological analyses were performed. Aliquots of 100 µL were serially diluted and spread plated on Rose-Bengal chloramphenicol agar. Agar plates were incubated at 22 ± 1 °C for 72 h; colonies were counted, and growth curves traced. The analyses were carried out in triplicate at five time points (d = day): d_0_, d_4_, d_7_, d_11_, d_14_.

Microscopic observations were conducted in triplicate on 1 mL pure culture of *G. sulphuraria* and *P. citrinum* and in co-culture. Fresh samples were deposited on a slide and immediately observed at microscope. The images were captured with a camera attached to an IBM computer running the Kontron Elektronik KS 300 image analysis system (Carl Zeiss MicroImaging s.p.a., Milan, Italy), for light and phase contrast microscopy analysis.

Data analyses were made using Sigmaplot 14 software. Results are presented as mean ± SD of three independent experiments. The statistical analysis was performed by one-way ANOVA with a Tukey *post-hoc* test to determine differences among the different cultures.

### GC–MS analysis of extracellular metabolites

For GC–MS metabolomic analysis we have employed for each culture type (i.e., *G. sulphuraria* pure culture; *P*. *citrinum* pure culture; co-culture of the alga plus fungus) three replicates. Cultures were prepared and grown as described above and each replicate was processed individually according to the following procedure.

After the 14 days incubation period, culture filtrates were obtained by filtering the cultures through sterile 0.45 µm cellulose membranes (Lifesciences) in a vacuum system. Each culture filtrate (0.8 L) was extracted with the same volume of ethyl acetate (EtOAc) for three times at the native pH (= 1.2). The organic phase was dried on Na_2_SO_4_ and evaporated under reduced pressure to give a yellowish oil residue. More abundant residues were obtained from co-cultures of alga plus fungus (≈ 120 mg) than from the pure alga (≈ 90 mg) or the pure fungus (≈ 15 mg) cultures. A stock solution (20 mg L^−1^) was prepared by dissolving appropriate amount of residue in chloroform: methanol (2:1). For GC–MS analysis, 100 µL of stock solution were transferred in a vial and dried with a stream of nitrogen at room temperature. The residue in the vial was derivatized with *N*,*O*-bis(trimethylsilyl)-trifluoroacetamide (BSTFA) (Fluka, Buchs, Switzerland) as previously described^62,63^ and then submitted to GC–MS analysis.

Trimethylsilyl derivatives were analysed by an Agilent 6850 GC (Milan, Italy), equipped with an HP-5MS capillary column (5% phenyl methyl poly siloxane stationary phase), coupled to an Agilent 5973 Inert MS detector operated in the full scan mode (*m*/*z* 29–550) at a frequency of 3.9 Hz and with the EI ion source and quadrupole mass filter temperatures kept, respectively, at 200 and 250 °C. Helium was used as carrier gas at a flow rate of 1 mL min^−1^. The injector temperature was 250 °C and the temperature ramp raised the column temperature from 70 to 280 °C: 70 °C for 1 min; 10 °C min^−1^ until reaching 170 °C; and 30 °C min^−1^ until reaching 280 °C. Then, it was held at 280 °C for 5 min. The solvent delay was 4 min.

For metabolomic profiling, a total of 18 GC–MS data files (comprising, for each culture type, two technical for each of the three biological replicates) were collected.

Raw GC–MS data were deconvoluted using the National Institute of Standards and Technology (NIST) program Automated Mass Spectral Deconvolution and Identification System (AMDIS)^35,64^, and then, ‘conserved metabolites’ across technical and biological replicates (conditions) were listed and tracked using SpectConnect program^36^.

SpectConnect matches components (metabolites) across observations on the basis of their chromatographic retention time and mass spectrum. By default, a metabolite whose chromatographic signal and mass spectrum persists in 75% of technical or biological replicates within one class or condition is designated by SpectConnect as a ‘conserved metabolite’. If observations belonging to several classes are presented to SpectConnect, a component is retained only if it is conserved in at least one class. Components which do not satisfy this condition are considered as accidents or artifacts of noise and are ignored. The main output of SpectConnect is a matrix, called RA matrix, which lists conserved metabolites (rows) and their relative abundances across all submitted observations (columns). In multiclass comparisons a set of logical values (the metabolite coordinates in the space of classes) defining for each metabolite the class or classes in which it is found to be conserved can also be deduced from the SpectConnect output.

The RA matrix created by SpectConnect was submitted to multivariate statistical analyses, such as Aggregative Hierarchical Clustering (AHC), Principal Component Analysis (PCA) and Partial Least-Squares Discriminant Analysis (PLS-DA), which were performed with our in-house.m script in MATLAB R2020b^65^ (Mathworks, Natick, MA, USA). Variables significantly contributing to the clustering and discrimination of samples were identified according to Variable Influence on Projection (VIP) values generated by PLS-DA processing. Before multivariate analysis the RA matrix was auto scaled in order to give equal weight to all predictor variables (metabolites). Our MATLAB.m script has also the logic to perform pairwise univariate analysis of data in the RA matrix. Univariate analysis compares metabolites in two different classes, one by one, and assigns to each metabolite a *p*-value calculated from a Student’s unpaired *t*-test and a fold change. In this work metabolites with *p-*value less than 0.05 and fold change greater than 2 or lower than 0.5 are considered to be differentially expressed in the two compared classes.

Metabolites were identified by comparing their EI mass spectra at 70 eV with spectra of known substances present in the NIST 20 mass spectral library^66^ and the Golm metabolome database^67,68^. Furthermore, the identification was supported by Kovats retention index (RI) calculated for each analyte by the Kovats equation, using the standard *n*-alkane mixture in the range C7-C40 (Sigma-Aldrich, Saint Louis, MO, USA)^69^.

## Supplementary Information

Supplementary Information.

## Data Availability

All relevant data are within the paper and its Supporting Information files.
