# Supplementary material for: Biological and metabolic effects of the association between the microalga Galdieria sulphuraria and the fungus Penicillium citrinum
Source: Sci Rep. 2023 Jan 31;13:1789. doi: 10.1038/s41598-023-27827-6 (PMC9889788; doi:10.1038/s41598-023-27827-6)
Supplement: Supplementary file 1 — Supplementary Information. [file 41598_2023_27827_MOESM1_ESM.pdf]

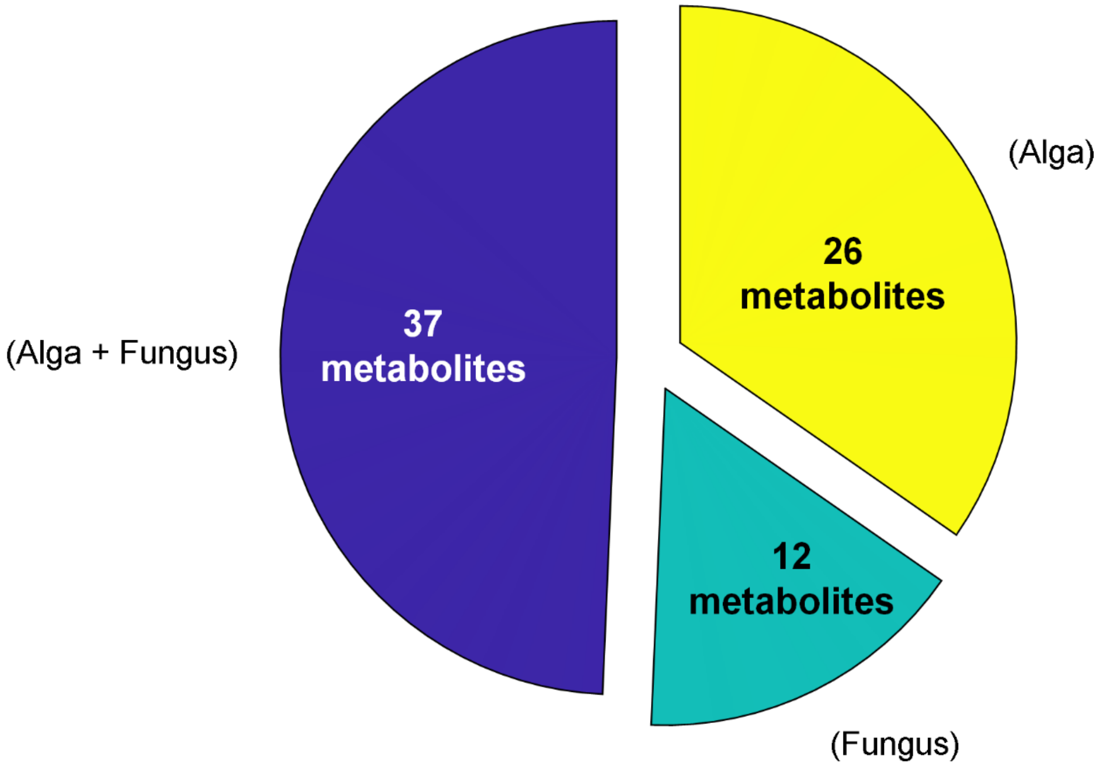

Fig S1. Number of metabolites detected in extracts from co-, pure Alga and pure Fungus cultures, respectively. (Alga) = *Galdieria sulphuraria* pure cultures; (Fungus) = *Penicillium citrinum* pure cultures; (Alga + Fungus) = *Penicillium citrinum* and *Galdieria sulphuraria* co-cultures.

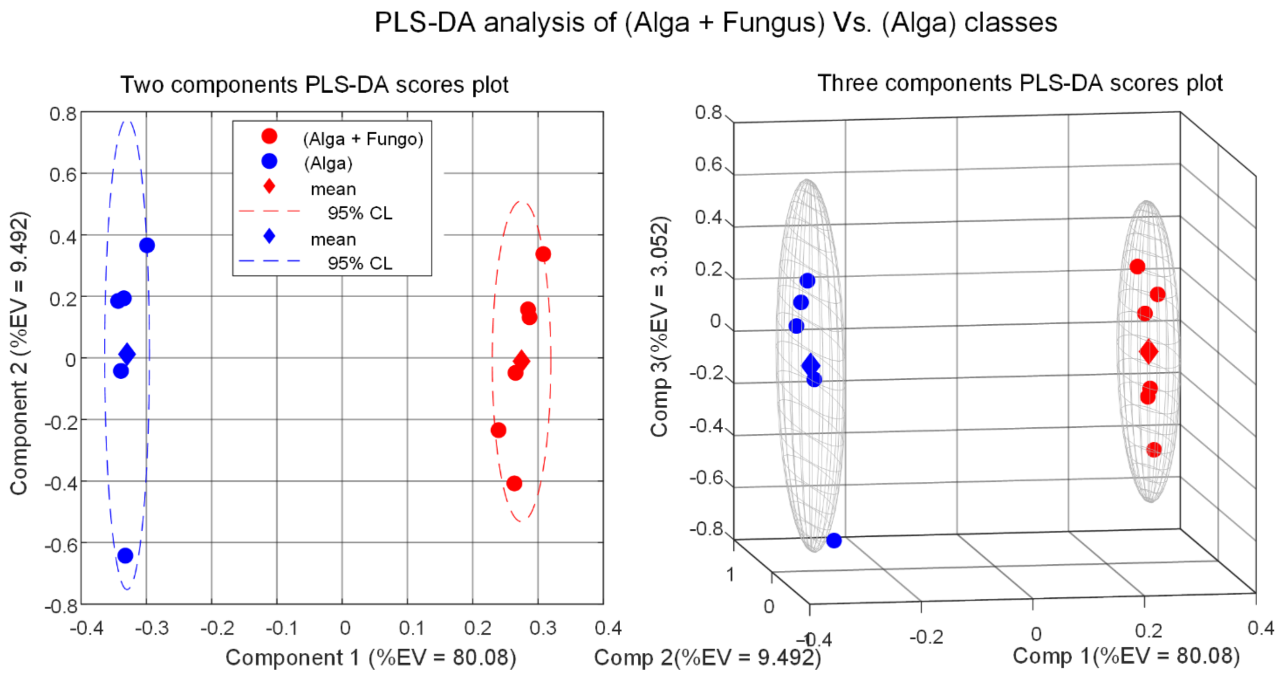

Fig S2. PLS-DA analysis of extracellular metabolites in cultures of (Alga + Fungus) and (Alga). (Alga) = *Galdieria sulphuraria* pure cultures; (Alga + Fungus) = *Penicillium citrinum* and *Galdieria sulphuraria* co-cultures.

Table S2. Results of univariate comparison between (Alga + Fungus) and (Alga) culture classes. VIP scores reported in column 2 have been obtained after PLS-DA analysis shown in Figure S2. Arrows indicate the direction (trend) of fold change comparing the two classes: (↑), upregulated in (Alga + Fungus); (↓), downregulated in (Alga + Fungus).

Name	VIP scores	*p*-values	FC: (Alga + Fungus) / (Alga)	Significance
(17)-Unknown (RI = 1470)	1.1173	2.2383E-12	4.5447 ↑	Yes
(21)-Tyrosol, 2TMS (RI = 1542)	1.1168	1.7679E-11	3.6192 ↑	Yes
(10)-Uracil, 2TMS (RI = 1357)	1.1163	4.6553E-11	78.9795 ↑	Yes
(33)-Protocatechoic acid, 3TMS (RI = 1838)	1.1143	6.8481E-08	2.9444 ↓	Yes
(11)-Fumaric Acid, 2TMS (RI = 1354)	1.1145	8.2027E-08	2.7882 ↑	Yes
(18)-CitraMalic acid, 3TMS (RI = 1491)	1.1146	9.8396E-08	38.1987 ↑	Yes
(38)-Unknown (RI = 1968)	1.0946	1.4977E-07	1.4878 ↓	No
(23)-2-IsopropylMalic acid, 3TMS (RI = 1597)	1.0902	5.205E-07	1.6203 ↓	No
(30)-Unknown (RI = 1705)	1.1046	1.0758E-06	8.2436 ↑	Yes
(19)-Malic acid, 3TMS (RI = 1507)	1.0758	1.8195E-06	1.2823 ↑	No
(36)-Unknown (RI = 1870)	1.0999	5.6242E-06	2.5989 ↓	Yes
(34)-Citric acid, (4TMS) (RI = 1845)	1.0986	0.000010922	29.7692 ↑	Yes
(6)- Isobutyric acid, 2TMS (RI = 1172)	1.0802	0.000037029	2.5606 ↑	Yes
(22)-Hydroxyglutaric acid, 3TMS (RI = 1589)	1.0773	0.000051833	2.2807 ↑	Yes
(8)-Glycerol, 3TMS (RI = 1287)	1.077	0.000054963	1.8165 ↑	No
(42)-Unknown (RI = 1860)	1.0952	0.00019091	29.7591 ↓	Yes
(4)-Lactic Acid, 2TMS (RI = 1084)	1.0899	0.00025087	3.0429 ↓	Yes
(35)-Homogentistic acid, 3 TMS (RI = 1855)	1.0539	0.00036279	5.31 ↑	Yes
(31)-Arabinofuranose,4TMS (RI = 1765)	1.0458	0.00048932	1.3709 ↓	No
(27)-Unknown (RI = 1628)	1.003	0.00066884	2.3651 ↓	Yes
(26)- α -Ketoglutaric acid, 3TMS (RI = 1623)	1.0474	0.00074545	2.8824 ↓	Yes
(43)-Palmitic acid, TMS (RI = 2049)	0.9587	0.0012	3.0527 ↓	Yes
(13)-Unknown (RI = 1391)	1.0389	0.0017	5.7951 ↑	Yes
(20)-Pyroglutamic acid, 2TMS (RI =)	0.99	0.0021	1.183 ↓	No
(16)-Unknown (RI = 1413)	1.0034	0.0023	1.8885 ↓	No
(12)-Unknown (RI = 1384)	0.9512	0.0025	1.2706 ↓	No
(29)- 4-Hydroxyphenylacetic acid, 2TMS (RI = 1653)	0.8976	0.0036	1.2174 ↓	No
(39)-Unknown (RI = 1367)	1.0132	0.0037	5.9558 ↑	Yes
(15)-Unknown (RI = 1402)	0.9749	0.0069	1.2369 ↓	No
(40)-meso-Erythritol, 4TMS (RI = 1532)	0.9622	0.0072	4.4343 ↑	Yes
(37)-4-HydroxyphenylLactic acid, 3TMS (RI = 1922)	0.9386	0.0114	4.5119 ↑	Yes
(25)-Glutaric acid, 3-hydroxy-3-methyl, 3TMS (RI = 1621)	0.8083	0.0122	6.3152 ↑	Yes
(14)-Unknown (RI = 1395)	0.9238	0.016	3.3819 ↑	Yes
(3)-2,3-Butanediol, 2TMS(RI = 1065)	0.8798	0.0171	1.5698 ↓	No
(7)-2-Propenoic acid, 3-methoxy-3-[(trimethylsilyl)oxy]-, methyl ester (RI = 1192)	0.8991	0.0172	1.5586 ↓	No
(1)-Boric acid, 3TMS (RI = 1021)	0.8547	0.0308	1.9104 ↓	No
(28)-Unknown (RI = 1643)	0.8365	0.0369	1.743 ↑	No
(5)-Sorbic acid, TMS (RI = 1135)	0.7466	0.0489	2.8781 ↑	Yes
(41)-Phosphoric acid,3TMS (RI = 1289)	0.6243	0.1197	3.5199 ↓	No

(9)-Succinic acid (2TMS)(RI = 1329)	0.4723	0.1887	1.235 ↑	No

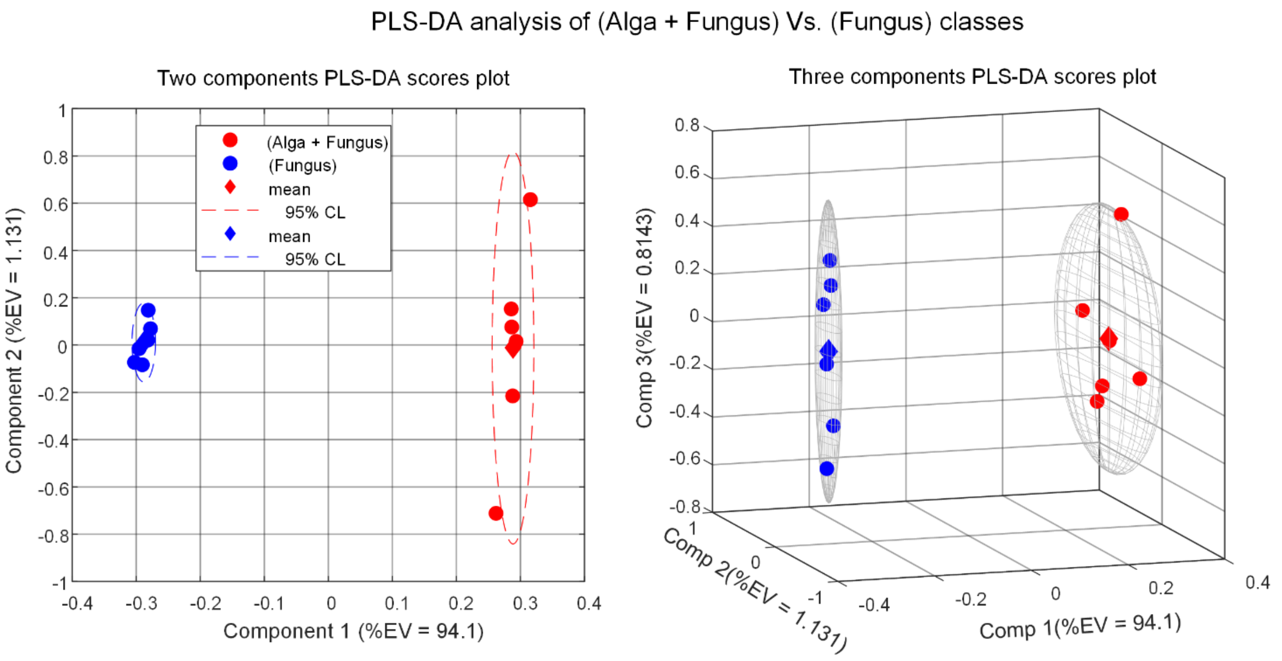

Fig S3. PLS-DA analysis of extracellular metabolites in cultures of (Alga + Fungus) and (Fungus). (Fungus) = *Penicillium citrinum* pure cultures; (Alga + Fungus) = *Penicillium citrinum* and *Galdieria sulphuraria* co-cultures.

Table S3. Results of univariate comparison between (Alga + Fungus) and (Fungus) culture classes. VIP scores reported in column 2 have been obtained after PLS-DA analysis shown in Figure S3. Arrows indicate the direction (trend) of fold change comparing the two classes: (↑), upregulated in (Alga + Fungus); (↓), downregulated in (Alga + Fungus).

Name	VIP scores	*p*-values	FC: (Alga + Fungus) / (Fungus)	Significance
(43)-Palmitic acid, TMS (RI = 2049)	1.0278	5.3786E-12	21.664 ↓	Yes
(21)-Tyrosol, 2TMS (RI = 1542)	1.0307	4.9548E-10	1335.4 ↑	Yes
(11)-Fumaric Acid, 2TMS (RI = 1354)	1.0307	7.7093E-10	216.350 ↑	Yes
(17)-Unknown (RI = 1470)	1.0305	1.8182E-09	428.587 ↑	Yes
(15)-Unknown (RI = 1402)	1.0304	2.5428E-09	291.561 ↑	Yes
(20)-Pyroglutamic acid, 2TMS (RI =)	1.0299	1.2287E-08	57.996 ↑	Yes
(28)-Unknown (RI = 1643)	1.03	1.3734E-08	103.481 ↑	Yes
(19)-Malic acid, 3TMS (RI = 1507)	1.0303	1.6054E-08	4997.9 ↑	Yes
(14)-Unknown (RI = 1395)	1.0296	2.1013E-08	148.312 ↑	Yes
(10)-Uracil, 2TMS (RI = 1357)	1.0297	2.2369E-08	491.772 ↑	Yes
(4)-Lactic Acid, 2TMS (RI = 1084)	1.0292	4.2786E-08	97.849 ↑	Yes
(18)CitraMalic acid, 3TMS (RI = 1491)	1.0284	1.1761E-07	62.859 ↑	Yes
(39)-Unknown (RI = 1367)	1.0284	1.468E-07	66.561 ↑	Yes
(38)-Unknown (RI = 1968)	1.0278	1.5398E-07	42.405 ↑	Yes
(33)-Protocatechoic acid, 3TMS (RI = 1838)	1.0279	1.5508E-07	38.734 ↑	Yes
(13)-Unknown (RI = 1391)	1.0273	2.2891E-07	32.490 ↑	Yes
(6)- Isobutyric acid, 2TMS (RI = 1172)	1.0273	2.5906E-07	1873.4 ↑	Yes
(31)-Arabinofuranose,4TMS (RI = 1765)	1.0175	3.0559E-07	10.966 ↑	Yes
(50)-Isovanillin, TMS (RI = 1554)	1.0257	4.9712E-07	13.577 ↓	Yes

(12)-Unknown (RI = 1384)	1.0261	5.2054E-07	52.563 ↑	Yes
(51)-Talofuranose (RI = 1873)	1.0255	8.2438E-07	80.197 ↓	Yes
(29)- 4-Hydroxyphenylacetic acid, 2TMS (RI = 1653)	1.0244	1.0578E-06	32.099 ↑	Yes
(7)-2-Propenoic acid, 3-methoxy-3-[(trimethylsilyl)oxy]-, methyl ester (RI = 1192)	1.0247	1.0716E-06	2153 ↑	Yes
(8)-Glycerol, 3TMS (RI = 1287)	1.0243	1.1662E-06	20.352 ↓	Yes
(37)-4-HydroxyphenylLactic acid, 3TMS (RI = 1922)	1.0237	1.4645E-06	43.914 ↑	Yes
(54)-Stearic acid, TMS (RI = 2245)	1.0227	1.5603E-06	9.037 ↓	Yes
(23)-2-IsopropylMalic acid, 3TMS (RI = 1597)	1.0247	1.5767E-06	42.342 ↑	Yes
(35)-Homogentistic acid, 3 TMS (RI = 1855)	1.0218	2.1756E-06	19.082 ↑	Yes
(30)-Unknown (RI = 1705)	1.0212	2.2953E-06	13.565 ↑	Yes
(40)-meso-Erythritol, 4TMS (RI = 1532)	1.0218	3.2077E-06	28.228 ↑	Yes
(52)-D-Glucose, 5TMS (RI = 1925)	1.0212	0.000003575	62.739 ↓	Yes
(36)-Unknown (RI = 1870)	1.0196	3.9921E-06	18.006 ↑	Yes
(22)-Hydroxyglutaric acid, 3TMS (RI = 1589)	1.0217	4.2293E-06	107.595 ↑	Yes
(3)-2,3-Butanediol, 2TMS(RI = 1065)	1.0171	7.2498E-06	1873.4 ↑	Yes
(16)-Unknown (RI = 1413)	1.0155	0.000010365	19.452 ↑	Yes
(34)-Citric acid, (4TMS) (RI = 1845)	1.0151	0.000010626	48.987 ↑	Yes
(44)-Glycolic acid, 2TMS (RI = 1091)	1.0133	0.000014478	40.384 ↓	Yes
(26)- α -Ketoglutaric acid, 3TMS (RI = 1623)	0.9998	0.000069463	77.511↑	Yes
(45)-Acetin,2TMS (RI = 1193)	0.9836	0.00017396	116.771 ↓	Yes
(5)-Sorbic acid, TMS (RI = 1135)	0.977	0.00028633	16.245 ↑	Yes
(9)-Succinic acid (2TMS)(RI = 1329)	0.9597	0.00049485	523.645 ↑	Yes
(27)-Unknown (RI = 1628)	0.8838	0.0043	87.346 ↑	Yes
(49)-Adipic acid, 2TMS (RI = 1515)	0.8676	0.0045	8.036 ↓	Yes
(47)-Unknown (RI = 1293)	0.8438	0.0065	1790.5 ↓	Yes
(25)-Glutaric acid, 3-hydroxy-3-methyl, 3TMS (RI = 1621)	0.8411	0.007	70.178 ↑	Yes
(1)-Boric acid, 3TMS (RI = 1021)	0.689	0.0364	1.824 ↓	No
